# Alterations in the Interleukin-1/Interleukin-1 Receptor Antagonist Balance Modulate Cardiac Remodeling following Myocardial Infarction in the Mouse

**DOI:** 10.1371/journal.pone.0027923

**Published:** 2011-11-28

**Authors:** Antonio Abbate, Fadi N. Salloum, Benjamin W. Van Tassell, Elena Vecile, Stefano Toldo, Ignacio Seropian, Eleonora Mezzaroma, Aldo Dobrina

**Affiliations:** 1 Victoria Johnson Research Laboratory and VCU Pauley Heart Center, Virginia Commonwealth University, Richmond, Virginia, United States of America; 2 School of Pharmacy, Virginia Commonwealth University, Richmond, Virginia, United States of America; 3 Department of Physiology and Pathology, University of Trieste, Trieste, Italy; South Texas Veterans Health Care System, United States of America

## Abstract

**Background:**

Healing after acute myocardial infarction (AMI) is characterized by an intense inflammatory response and increased Interleukin-1 (IL-1) tissue activity. Genetically engineered mice lacking the IL-1 receptor (IL-1R1-/-, not responsive to IL-1) or the IL-1 receptor antagonist (IL-1Ra, enhanced response to IL-1) have an altered IL-1/IL-1Ra balance that we hypothesize modulates infarct healing and cardiac remodeling after AMI.

**Methods:**

IL-1R1-/- and IL-1Ra-/- male mice and their correspondent wild-types (WT) were subjected to permanent coronary artery ligation or sham surgery. Infarct size (trichrome scar size), apoptotic cell death (TUNEL) and left ventricular (LV) dimensions and function (echocardiography) were measured prior to and 7 days after surgery.

**Results:**

When compared with the corresponding WT, IL-1R1-/- mice had significantly smaller infarcts (−25%), less cardiomyocyte apoptosis (−50%), and reduced LV enlargement (LV end-diastolic diameter increase [LVEDD], −20%) and dysfunction (LV ejection fraction [LVEF] decrease, −50%), whereas IL-1Ra-/- mice had significantly larger infarcts (+75%), more apoptosis (5-fold increase), and more severe LV enlargement (LVEDD increase,+30%) and dysfunction (LVEF decrease, +70%)(all P values <0.05).

**Conclusions:**

An imbalance in IL-1/IL-1Ra signaling at the IL-1R1 level modulates the severity of cardiac remodeling after AMI in the mouse, with reduced IL-1R1 signaling providing protection and unopposed IL-1R1 signaling providing harm.

## Introduction

Healing and cardiac remodeling after acute myocardial infarction (AMI) are characterized by an intense inflammatory response within the myocardium. Interleukin-1β (IL-1β) is a potent pro-inflammatory mediator with local and systemic effects mediated by the IL-1 type I receptor (IL-1R1), the only signaling membrane receptor for IL-1β [1]. Genetically engineered mice lacking the gene encoding for the IL-1 type I receptor (IL-1R1-/- mice) are not responsive to IL-1β or IL-1α, but have an otherwise normal phenotype and remain responsive to other pro-inflammatory stimuli such as lipopolysaccharide (LPS) and Tumour Necrosis Factor-α [1]–[2].

Interleukin-1 receptor antagonist is a natural occurring protein that competitively inhibits IL-1β (and IL-1α) signalling, but fails to recruit the IL-1 receptor associated protein and therefore does not transduce signal [1]–[3]. Mice with genetic deletion of the gene encoding for IL-1Ra (IL-1Ra-/-mice) have enhanced response to IL-1, are more susceptible to Listeria monocytogenis infections, and may develop spontaneously occurring inflammatory arthritis and arteritis [4]–[6]. However, no gross cardiac abnormalities have been reported in these mice.

In many conditions, the balance between IL-1β and IL-1Ra at the receptor level determines the inflammatory activity and severity of the disease [3], [7]. IL-1 is known to influence ischemia, heart failure, and cardiac remodeling after acute myocardial infarction in the mouse [8]–[10]. In order to demonstrate the pivotal role of IL-1 signaling on cardiac remodeling after AMI, we studied two independent strains of mice with genetic modifications that alternatively enhanced IL-1 signaling (IL-1Ra deletion, IL-1Ra-/-) or suppressed IL-1 signaling (IL-1R1-/-) in a model of severe ischemic cardiomyopathy due to permanent coronary artery ligation.

## Methods

### Experimental design

IL-1R1-/- male mice (strain B6.129S1-*Il1r1tm1Roml*/J) and the corresponding wild-type adult male mice (strain B6.129SF2J) were purchased from Jackson Laboratory (Maine, USA)[11].

IL-1Ra-/- male mice (strain B6.129S-IL-1RNtm1Dih/J) were also purchased from Jackson Laboratory [12] and wild-type adult male mice (strain C56BL/6J) were used as recommended by the supplier in consideration of the backcrossing of the B6-129 generated mouse into the C56BL line [12].

All animal experiments were conducted under the guidelines on humane use and care of laboratory animals for biomedical research published by National Institutes of Health (No. 85-23, revised 1996). The study was approved by the Institutional Animal Care and Utilization Committee (IACUCU) of the Virginia Commonwealth University (AM20114). Mice were received at variable ages. Upon their arrival, all animals were allowed to readjust to the housing environment for at least 7 days before any experiment, with freely accessible standard rodent food and water. All mice were between 14 and 16 weeks at time of surgery.

AMI by permanent left coronary artery ligation was induced in 4 groups of mice (IL-1R1-/- and B6.129 WT mice; IL-1Ra-/- and C57Bl WT mice)(N = 10–12 per group), while 4 additional groups of mice (N = 5) underwent a sham operation including every step except the coronary artery ligation. The mice were then allowed to recover for 7 days.

### Surgical procedure

The animals under anesthesia (pentobarbital 50–70 mg/Kg) were orally intubated, and then underwent opening of the chest and ligation of the left coronary artery (or sham surgery, in which the needle was passed around the artery but it was not ligated), as previously described [8].

### Histology

For immunofixation, animals were sacrificed at 7 days and the heart was then mounted on a Langendorff apparatus. The coronary arteries were perfused with 10% formalin and subsequently the whole heart was submersed in the same solution. After fixation, the mid-ventricular section of each heart was cut (approx. 3 mm thickness) and then the samples were dehydrated and included in paraffin.

The infarct scar size was measured as scar area in sections stained with Masson's trichrome (Sigma-Aldrich, St Louis, Mo) by computer morphometry using a BIOQUANT imaging software and expressed as a percentage of the whole left ventricle mid-ventricular section.

Nuclear DNA fragmentation (terminal deoxynucleotidyl transferase-mediated dUTP nick-end labeling–ApopTag, Chemicon) was used as a marker for apoptosis. Only cardiomyocytes (cardiac actin+) expressing TUNEL staining in the nucleus were counted in the peri-infarct area, defined as the zone bordering the infarct where viable myocardium was prevalent and reparative fibrosis was only marginal, and in the remote unaffected myocardium. The apoptotic rate (AR) is expressed as the number of apoptotic cardiomyocytes on all cardiomyocytes per field, analysing 5 of more fields covering the entire infarct and border zones in the mid-ventricular section. The pathologist performing the count was unaware of the mouse genotype or surgical procedure performed.

### Echocardiography

Transthoracic echocardiography was performed before surgery and 7 days later, using the Vevo770™ imaging system (VisualSonics Inc., Toronto, Canada), equipped with a 30 MHz probe, as previously described [8]. Briefly, the heart was imaged in the short-axis view of the LV. The M-mode cursor was positioned perpendicular to the anterior wall in order to measure left ventricular end-diastolic and end-systolic diameters (LVEDD and LVESD, respectively) at the level of the papillary muscles below the mitral valve tip. LV ejection fraction (LVEF) and mass were calculated. The investigator performing and reading the echocardiogram was blinded to the mouse genotype and surgical procedure performed.

### Statistical analysis

Data are presented as mean and standard error of the mean. Comparisons between the genetically modified mice (IL-1R1-/- or IL-1Ra-/-) and the corresponding WT at a single time point was performed using the Student T test for unpaired data. In order to compare changes over time between 2 groups, ANOVA for repeated measures was using analyzing the effect of time_x_interaction. SPSS 16.0 package was used. Two-tail significance was set at 0.05.

Direct comparison between the IL-1R1-/- and IL-1Ra-/- mice was not possible considering that 2 strains had different genetic background (B6.129 and C57Bl, respectively), therefore in order to visually compare the IL-1R1-/- and IL-1Ra-/- mice, the values of LVEDD, LVESD, LVEF, infarct size and apoptosis was normalized to the corresponding WT, and expressed as% of WT.

## Results

### Cardiac morphology

**Figure 1:**
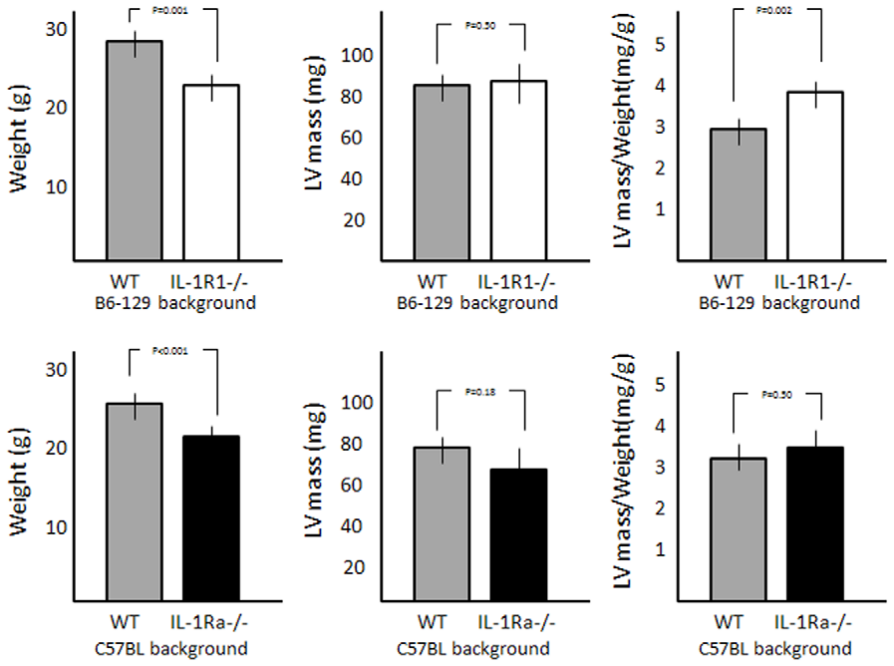
Body weight and left ventricular mass in IL-1R1-/- and IL-1Ra-/- mice. Genetically modified mice (both IL-1R1-/- and IL-1Ra-/- ) have significantly smaller body weights compared with the respective wild-type animals (WT). Left ventricular (LV) mass calculated at echocardiography was not statistically different comparing IL-1R1-/- and IL-1Ra-/- and respectively WT, but the IL-1R1-/- had significantly greater LV mass/weight ratio. N = 8–10 per group.

IL-1R1-/- mice had significantly smaller body weight but similar LV mass reflecting a significantly increased LV mass to body weight ratio (Figure 1). IL-1Ra-/- mice also had significantly smaller body weight, but similar LV mass and similar LV mass to body weight ratio (Figure 1).

### Survival after surgery

Two of 12 (17%) of IL-1R1-/- mice and 5 of 14 (36%) of the corresponding WT mice died after AMI surgery. Eight of 15 (54%) IL-1Ra-/- mice died after coronary artery ligation surgery compared to 4 of 12 (33%) of the corresponding WT mice. Free wall cardiac rupture was found in 2 IL-1Ra-/- mice (13%), none of the IL-1R1-/- mice, and none of the WT mice. None of the sham operated animals died. None of the differences reached the statistical significance set at a P of 0.050.

### Infarct scar size

**Figure 2:**
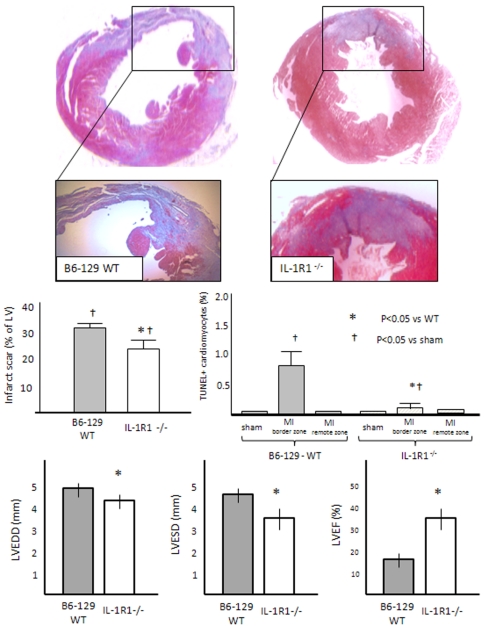
Infarct size, apoptosis and left ventricular remodeling in wild-type and IL-1R1-/- mice. When compared with the corresponding wild-type mice, infarct scar size (Masson's trichrome stain) and cardiomyocyte apoptotic rate (in-situ end labelling of DNA fragmentation–TUNEL) were significantly smaller in IL-1R1-/- mice. Moreover, IL-1R1-/- mice had less left ventricular enlargement and systolic dysfunction 1 week after AMI. N = 6–8 per group.

**Figure 3:**
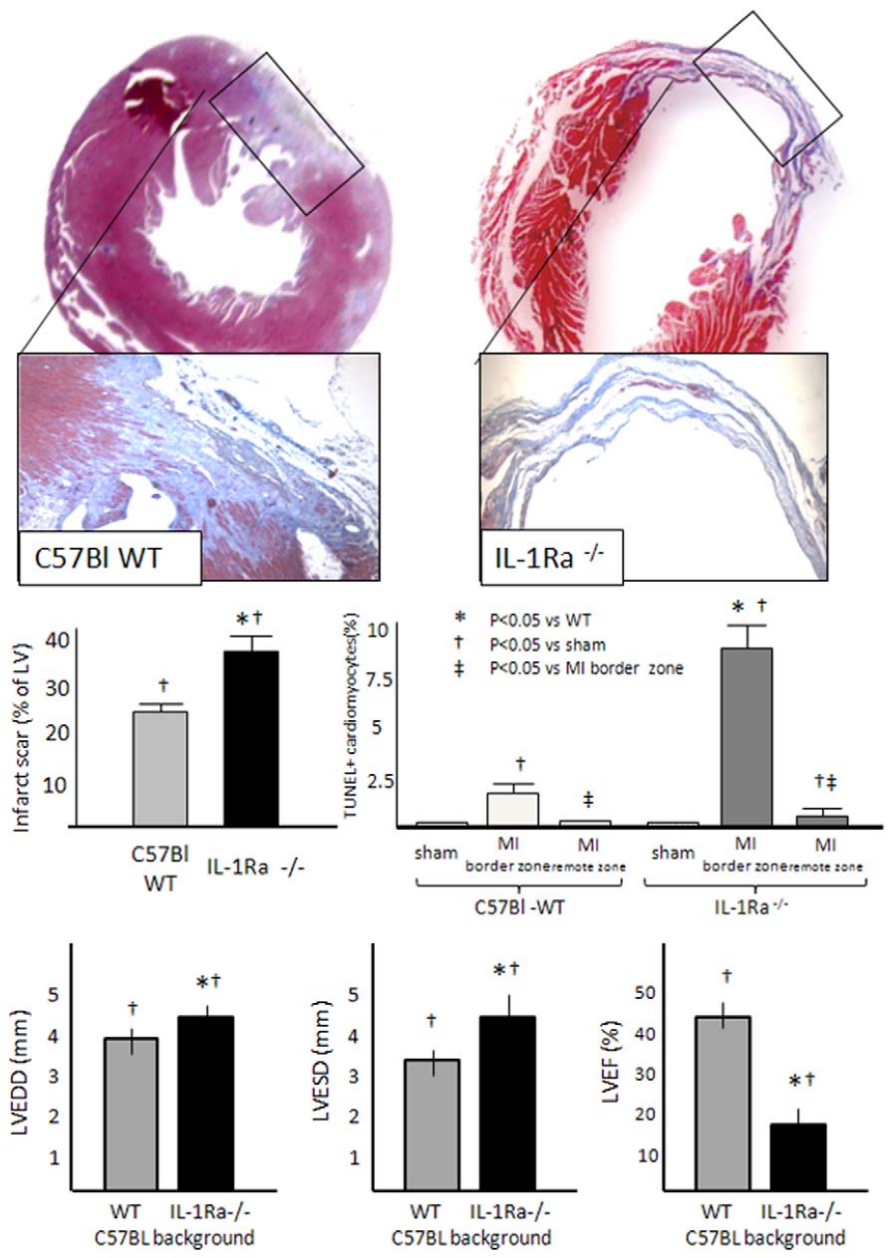
Infarct size, apoptosis and left ventricular remodeling in wild-type and IL-1Ra-/- mice. When compared with the corresponding wild-type mice, infarct scar size (Masson's trichrome stain) and cardiomyocyte apoptotic rate (in-situ end labelling of DNA fragmentation–TUNEL) were significantly greater in IL-1Ra-/- mice. Moreover, IL-1Ra-/- mice had less left ventricular enlargement and systolic dysfunction 1 week after AMI. N = 6–8 per group.

Infarct scar size was measured 7 days after AMI surgery. Figures 2 and 3 show representative images from the AMI groups for the IL-1R1-/- and IL-1Ra-/-, respectively. When compared with the corresponding WT mice, IL-1R1-/- mice had a significantly smaller infarct size, whereas IL-1Ra-/- had a significantly larger infarct size. Sham operated mice had no measurable infarct (data not shown).

### Cardiomyocyte apoptosis

Cardiomyocyte apoptosis was significantly greater in the peri-infarct myocardium versus the remote myocardium and versus the anterior wall in sham operated animals in all 4 groups. When compared with the corresponding WT mice, IL-1R1-/- mice had significantly less cardiomyocyte apoptosis in the peri-infarct myocardium (Figure 2), whereas IL-1Ra-/- mice had significantly more cardiomyocyte apoptosis both in the peri-infarct and remote myocardium (Figure 3).

### Left ventricular enlargement and systolic dysfunction

AMI lead to LV enlargement and systolic dysfunction versus sham operated mice (LVEDD, LVESD and LVEF–P<0.001). When compared with the corresponding WT mice, IL-1R1-/- mice had significantly less LV enlargement (measured as LVEDD and LVESD) and less LV systolic dysfunction (measured as LVEF)(Figure 2), whereas IL-1Ra-/- mice had worse LV enlargement and dysfunction (Figure 3).

### Indirect comparison between IL-1R1-/- and IL-1Ra-/- mice

**Figure 4:**
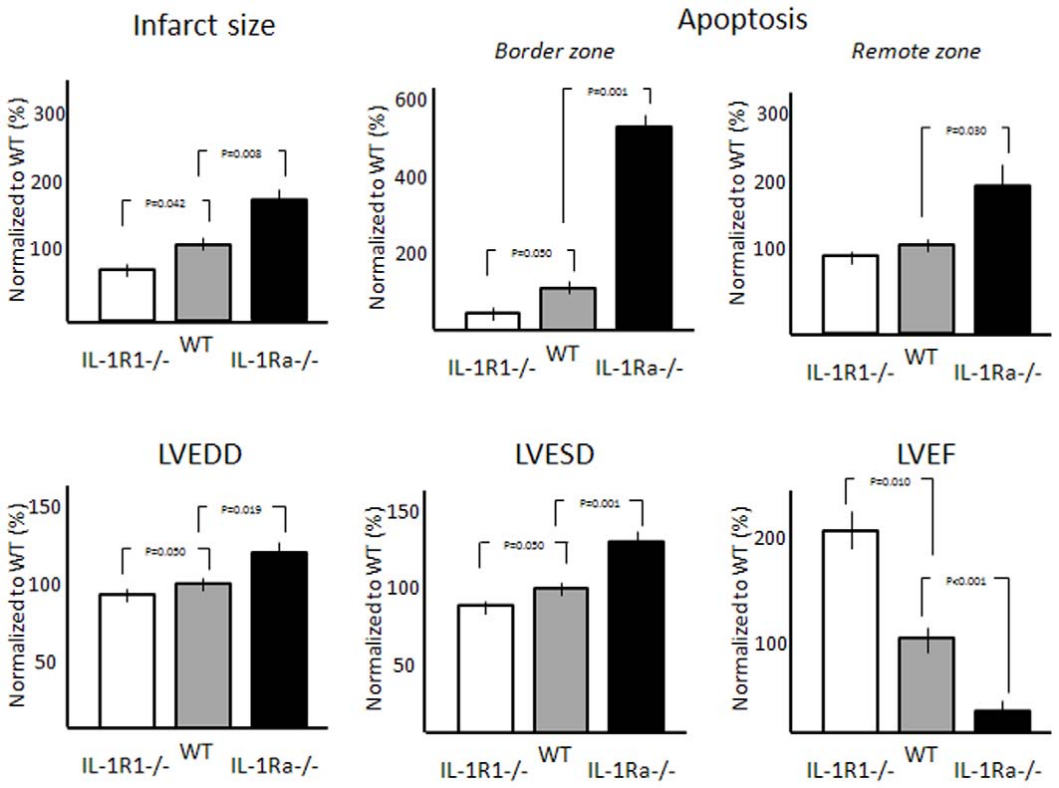
Indirect comparison between the IL-1R1-/- and the IL-1Ra-/-. Considering that IL-1R1-/- and the IL-1Ra-/- mice were generated in different backgrounds, a direct comparison between the 2 strains cannot be made. The Figure shows an indirect comparison between the 2 strains in which the value of each strain is normalized to the corresponding wild-type mouse. In the comparison, the IL-1R1-/- mouse shows to have smaller infarcts, less apoptosis and more favorable remodeling, wheras the IL-1Ra-/- mouse shows large infarcts, more apoptosis, and more unfavorable remodeling.

When data from IL-1R1-/- and IL-1Ra-/- mice were normalized to the WT, the WT mice displayed an intermediate phenotype between the IL-1R1-/- mice–which were protected–and the IL-1Ra-/- mice in which heart damage was exacerbated (Figure 4).

## Discussion

IL-1 is considered a key inflammatory mediator in the response to sterile inflammation such as acute myocardial infarction. By using 2 genetically modified mouse strains, the current study shows that the lack of IL-1R1 signaling or the unopposed IL-1R1 signaling (due to lack of the IL-1Ra) is sufficient to modulate the natural course of post-AMI cardiac remodeling.

In this mouse model of severe ischemic cardiomyopathy, the lack of signaling through the IL-1R1 signaling post-AMI is associated with a more favorable remodeling. This is an agreement with a prior study showing more favorable remodeling following myocardial ischemia-reperfusion injury due to transient coronary occlusion [13].

The results of these experiments also show that endogenous IL-1Ra plays a protective role in the myocardium during AMI by preventing cell death and ensuing adverse cardiac remodeling. While the data presented are in accordance with the proposed cardioprotective effects of IL-1Ra in myocardial ischemia and infarction [8], [14], the current study shows for the first time that the lack of endogenous IL-1Ra leads to more adverse remodeling. Previous experiments showed that IL-1Ra transgenic mice, in which IL-1Ra is overexpressed in fibroblasts, subjected to global cardiac ischemia-reperfusion surgery simulating heart transplantation had a smaller extent of subendocardial infarct and reduced apoptosis in cardiomyocytes [14]. Moreover, supplementation of recombinant human IL-1Ra to the mouse lead to improved cardiac remodeling after AMI [8].

These findings reinforce the concept that modulation of IL-1 is a viable target for interventions aimed at preventing heart failure after AMI, and, within the challenges and limitations of preclinical and translational studies [15], they provide the frame to interpret studies using pharmacologic IL-1 blockers in patients with AMI [16]. With the exception of one study using a hamster anti-mouse IL-1β antibody (not commercially available) which showed adverse remodeling and increased cardiac rupture [17], all other studies using either a recombinant human IL-1Ra [8], a recombinant fusion protein binding circulation IL-1β and IL-1α (IL-1Trap)[9], or a mouse engineered anti-IL-1β antibody (XMA052 MG1K)[10], have shown a more favorable remodeling associated with IL-1 blockade and no increase in the incidence of cardiac rupture. All together, the data deriving from the genetically manipulated mice and the more recent studies using IL-1 blockers argue against the concept that IL-1β is necessary for infarct healing.

During AMI, tissue injury leads to an intense sterile inflammation that is likely to be initially induced by toll-like receptor (TLR) signaling by the many danger-associated molecular patterns generated during cell death. TLR agonists induce a powerful activation of nuclear factor κ-B and induction of numerous inflammatory mediators that recruit and activate leukocytes within the injured myocardium. IL-1 is certainly among these soluble mediators that greatly amplifies the inflammatory response in a paracrine and autocrine fashion (“IL-1 induces IL-1”)[18]. Pharmacologic IL-1 blockade (or genetic deletion of the IL-1R1) does not impede TLR signaling allowing for the initial inflammatory response to injury, but may limit the occurrence of additional tissue injury induced by IL-1. Cardiomyocytes in the border zones of the AMI synthesize IL-1Ra to protect themselves from IL-1 mediated injury. Mice lacking IL-1Ra lack this endogenous protective mechanism and hence their cardiomyocytes (and other cell types) are at higher risk of death. A clinical syndrome in children characterized by life-threatening systemic inflammation (with particular involvement of skin and bone involvement) has been linked to the genetic deficiency of the IL-1Ra (DIRA), and has been found to be responsive to exogenous IL-1Ra [19]. The DIRA syndrome, in which endogenous IL-1Ra is lacking, is similar to syndromes of enhanced IL-1β production due to genetic alterations of the cryopyrin gene (Cryopyrin-associated periodic syndromes [CAPS]), which are also responsive to exogenous IL-1Ra [20], thus confirming the key role of IL-1/IL-1Ra balance in disease activity.

One major limitation of the current study is that the IL-1R1-/- and IL-1Ra-/- strains were generated in 2 different mouse lines and hence a direct comparison between the 2 mutants cannot be performed. Indeed, mouse strain may determine significant differences in the outcome of wound healing after myocardial infarction [21]. With the use of indirect comparison after normalization to the respective wild-type mouse, we could show that a gradient of severity was present going from the IL-1R1-/- mice through the WT and to the IL-1Ra-/- mice. Moreover, the WT used in each study were not littermates because not commercially available.

The relatively small number of mice studies makes it unable to have reliable data on survival and on cardiac rupture. We noticed a small increase in cardiac rupture in IL-1Ra-/- mice but it did not reach statistical significance. Moreover, the lack of data regarding leukocyte density and activation and MMP activation does not allow us to determine whether enhanced MMP activation in IL-1Ra-/- is the cause of cardiac rupture.

Finally, no data on right ventricular dimensions, function or pathology was recorded. Defining whether IL-1 is involved in biventricular remodeling [22]–[23] could have further improved our understanding of heart failure.

The mechanisms by which IL-1 signaling at the IL-1R1 level drives adverse cardiac remodeling is not completely clear and have not been explored in the current study. In vitro, IL-1 signaling modulates cardiomyocyte survival independent of the function of leukocytes [8], [24]. IL-1 upregulates caspase-1 activity which has been shown to promote cell death in cardiomyocytes [24]–[27]. In vitro, IL-1Ra has been shown to promote cell survival and to inhibit caspase-1 and caspase-9 [8]. Whether IL-1Ra promotes cell survival in ways other than competing with IL-1β at the IL-1R1 levels is unknown, and requires further studies.

We chose the model of non-reperfused AMI because although most of the patients with AMI receive some form of intervention aimed at obtaining reperfusion, presently many patients achieve incomplete tissue level reperfusion (no reflow), which negates the benefit of reperfusion and is associated with an even greater risk of subsequent heart failure [28]–[29].

In conclusion, modification of IL-1 signaling determining an imbalance between IL-1 and IL-1Ra at the IL-1R1 level determines the severity of cardiac remodeling after AMI in the mouse, with reduced IL-1R1 signaling providing protection and unopposed IL-1R1 signaling providing harm.
